# Transcriptome network of the papillary thyroid carcinoma radiation marker CLIP2

**DOI:** 10.1186/s13014-020-01620-5

**Published:** 2020-07-29

**Authors:** Martin Selmansberger, Agata Michna, Herbert Braselmann, Ines Höfig, Kenji Schorpp, Peter Weber, Natasa Anastasov, Horst Zitzelsberger, Julia Hess, Kristian Unger

**Affiliations:** 1 Research Unit Radiation Cytogenetics, Helmholtz Zentrum München, German Research Center for Environmental Health GmbH, 85764 Neuherberg, Germany; 2 Institute of Radiation Biology, Helmholtz Zentrum München, German Research Center for Environmental Health GmbH, 85764 Neuherberg, Germany; 3 Institute for Molecular Toxicology and Pharmacology, Helmholtz Zentrum München, German Research Center for Environmental Health GmbH, 85764 Neuherberg, Germany; 4 Department of Radiation Oncology, University Hospital, LMU Munich, Munich, Germany; 5 Clinical Cooperation Group ‘Personalized Radiotherapy in Head and Neck Cancer’, Helmholtz Zentrum München, German Research Center for Environmental Health GmbH, 85764 Neuherberg, Germany

## Abstract

**Background:**

We present a functional gene association network of the CLIP2 gene, generated by *de-novo* reconstruction from transcriptomic microarray data. CLIP2 was previously identified as a potential marker for radiation induced papillary thyroid carcinoma (PTC) of young patients in the aftermath of the Chernobyl reactor accident. Considering the rising thyroid cancer incidence rates in western societies, potentially related to medical radiation exposure, the functional characterization of CLIP2 is of relevance and contributes to the knowledge about radiation-induced thyroid malignancies.

**Methods:**

We generated a transcriptomic mRNA expression data set from a CLIP2-perturbed thyroid cancer cell line (TPC-1) with induced CLIP2 mRNA overexpression and siRNA knockdown, respectively, followed by gene-association network reconstruction using the partial correlation-based approach *GeneNet*. Furthermore, we investigated different approaches for prioritizing differentially expressed genes for network reconstruction and compared the resulting networks with existing functional interaction networks from the Reactome, Biogrid and STRING databases. The derived CLIP2 interaction partners were validated on transcript and protein level.

**Results:**

The best reconstructed network with regard to selection parameters contained a set of 20 genes in the 1st neighborhood of CLIP2 and suggests involvement of CLIP2 in the biological processes DNA repair/maintenance, chromosomal instability, promotion of proliferation and metastasis. Peptidylprolyl Isomerase Like 3 (PPIL3), previously identified as a potential direct interaction partner of CLIP2, was confirmed in this study by co-expression at the transcript and protein level.

**Conclusion:**

In our study we present an optimized preselection approach for genes subjected to gene-association network reconstruction, which was applied to CLIP2 perturbation transcriptome data of a thyroid cancer cell culture model. Our data support the potential carcinogenic role of CLIP2 overexpression in radiation-induced PTC and further suggest potential interaction partners of the gene.

## Background

Thyroid cancer incidence rates worldwide are among the fastest rising over the last decades, thus becoming the most common endocrine malignancy [1]. While environmental factors, lifestyle factors, medical radiation-exposure and the effect of overdiagnosis due to improved screening methods are discussed, their impact on the increased incidence rates remains to be proved [2]. Exposure to ionizing radiation at young age, both internal and external, is a well-known risk factor for the development of thyroid carcinoma [3]. The consequences of the Chernobyl nuclear accident enabled comprehensive studies of the epidemiologic effects of exposure to ionizing radiation and molecular characteristics of radiation induced malignancies. The results of these studies provide a valuable basis for current and future studies on radiation-associated thyroid carcinomas such as secondary cancers due to medical radiation exposure [4, 5]. In particular, just a few years after the Chernobyl accident, an increased papillary thyroid carcinoma (PTC) incidence was observed in young children exposed to radiation related to a thyroid radiation dose mainly due to ingested iodine-131 [6–12]. The thyroid doses of the 13,000 subjects of the Ukrainian-American (UkrAm) due to ingestion of I-131 were estimated to average 0.66 Gy [13]. The availability of tissue samples through the Chernobyl Tissue Bank promoted a huge number of molecular studies including investigations of specific RET/PTC gene rearrangements (reviewed in [14]), radiation-associated gene alterations as determined by global gene expression analyses and global genomic copy number analysis [15–21]. An in-depth genomic copy number analysis of closely matched exposed and non-exposed PTC patients revealed a radiation-specific DNA gain of chromosome band 7q11.22–11.23 and overexpression of the CLIP2 gene in the exposed group, which was subsequently validated at the protein level [22, 23]. Additionally, a positive dose response for the probability of CLIP2 overexpression in PTCs in exposed children younger than five was observed [23]. Further, the CLIP2 radiation marker has the potential to be implemented in the diagnosis of secondary thyroid carcinomas as consequence of medical exposure to ionizing radiation e.g. during radiologic imaging or radiotherapy [24]. The doses due to adverse radiation of non-target organs during radiotherapy were measured to be in the range of the investigated post-Chernobyl PTC and thereby promote the relevance of the derived results for medical radiation exposure and its adverse effects [25].

In the present study we aimed to further characterize the function of CLIP2 at the transcriptional level. Gene association networks (GANs) were reconstructed using transcriptome data from the thyroid cancer cell culture model TPC-1 after CLIP2 siRNA knockdown or stable CLIP2 overexpression. In order to identify the best approach for the preselection of genes subjected to GAN reconstruction, we compared the resulting networks to established and published known interaction networks. In order to gain deeper insights into the functional role of CLIP2, we analyzed the resulting network for possible interaction partners by identification of the first (i.e. direct) neighbors of CLIP2. Amongst these was the Peptidylprolyl Isomerase Like 3 (PPIL3) gene, which we already identified as possible functional interaction partner of CLIP2 in a reconstructed GAN from patient tissue samples and which we validated by protein co-expression analysis [22].

## Material and Methods

### Cloning of CLIP2

CLIP2 was amplified from TPC-1 cDNA using gene specific primers (Forward: 5′-TCTAGAGAATTCGCCACCATGCAGAAGCCCAGCGGCCTGAAG-3′; Reverse: 5′.

TCTAGATAGCGGCCGCGTGCTTGTCCTCTTGTTTCTGAGC-3′) and cloned into the lentiviral vector pCDH-EF1-MCS-T2A-copGFP (System Biosciences, Mountain View, CA, USA). The sequence of the inserted CLIP2 fragment was confirmed by sequencing (NCBI reference sequence: NM_032421.2).

### Lentivirus production and infection of TPC-1 cells

Replication-defective lentiviral virions were produced by transient co-transfection of HEK293T cells with pCDH-EF1-CLIP2-T2A-copGFP or control vector pGreenPuro (System Biosciences, Mountain View, USA) and packaging plasmids (pRSV.Rev., pMDLg/pRRE, pMD2.G) as previously described [26, 27]. TPC-1 cells (2 × 10^5^ cells/6-well, seeded 1 day prior) were infected with lentivirus containing medium (MOI up to 30, multiplicity of infection, i.e. the number of infectious particles/target cell) as previously described [28]. Three days after infection, GFP-expression was monitored and single cell sub-cloning was performed.

### Cultivation of TPC-1 cells and siRNA knockdown of CLIP2 mRNA

Cell line identity of parental TPC-1 cells and all clones derived by lentivirus treatment was validated by STR-typing (SI Table 1). The TPC-1 cells were cultivated in RPMI medium (with 10% FCS, 1% PenStrep) at 37 °C and 5% CO_2_. The (forward) transfection of the TPC-1 cells with the CLIP2 siRNA was carried out in 6 well plates at 60–80% confluence with antibiotic-free medium. The siRNA-Lipofectamine complex was generated according to the manufacturers protocol using the Lipofectamine RNAiMAX Transfection Reagents (Life Technologies). The highest transfection efficiency with > 97% of transfected cells was determined in a separate experiment, with 75 pmol of Block-it Alexa Fluor Red siRNA and 7.5 μl Lipofectamin. The fraction of transfected cells was determined by FACS analysis. At the time of transfection 2.5 ml of antibiotic-free cell culture medium was present in the in the well and 250 μl of siRNA-Lipofectamine complex was added (75 pmol siRNA, 7.5 μl Lipofectamine).

### Cultivation and harvesting of siRNA perturbed TPC-1 cells

After the removal of the medium containing the transfection complex after 5 h, the cells were cultivated in RPMI medium (with 10% FCS, 1% PenStrep) at 37 °C and 5% CO_2_. The cells were harvested after 20, 28, 44, 48, 52, 68, 72, and 76 h after transfection in the first knockdown (KD) experiment and after 48 and 72 h in the second knockdown experiment after transfection using a cell scraper and immediately stored at − 80 °C as cell pellet. The second KD experiment included non-transfected and non-sense siRNA transfected cells in order to reveal genes, which are differentially expressed due to the transfection procedure/chemicals.

### RNA isolation

Total RNA was isolated from TPC-1 cell pellets using the RNeasy Mini Kit (Qiagen, Hilden, Germany). RNA-quality was assessed using an Agilent 2100 Bioanalyzer (Agilent Technologies).

### Quantitative RT-PCR (qRT-PCR)

Reverse transcription was performed using the QuantiTect Reverse Transcription Kit (Qiagen, Hilden, Germany). qRT-PCR reactions (10 μl) were carried out in triplicates in a ViiA 7 Real Time PCR System in combination with the ViiA 7 Software v1.2.2 (Life Technologies, Carlsbad, USA). TaqMan gene expression assay Hs00185593_m1 was used for analyzing the CLIP2 gene, whereas assays for ACTB (Hs99999903_m1), B2M (Hs99999907_m1), and PGK1 (Hs99999906_m1) were used for endogenous normalization. Relative expression levels were calculated using the Delta-Delta Ct method [29]. TaqMan probes used for in situ network validation are listed in SI Table 2.

### Verification of CLIP2 knockdown by Western blot

CLIP2 knockdown on protein level was verified using Western Blot analysis. Cells were washed once with cold 1xPBS and lysed using RIPA lysis buffer containing protease (Complete; Roche, Mannheim, Germany) and phosphatase inhibitor cocktails (PhosStop; Roche, Mannheim, Germany). 20 μg (knockdown) or 10 μg (overexpression) of the whole cell lysate was subjected to 10% SDS-PAGE and transferred to a PVDF membrane (Immobilon-P; Millipore, Billerica, MA, USA). Roti-Block reagent (Carl Roth, Karlsruhe, Germany) was used for blocking. The membranes were incubated with anti-CLIP2 polyclonal rabbit antibody (1:1000; Santa Cruz Biotechnology, Dallas, TX, USA). After incubation with the corresponding secondary antibody signals were visualized with the enhanced chemiluminescence system (Amersham ECL Plus Western blotting detection system; GE Healthcare, Chalfont St. Giles, UK).

### Microarray based genome-wide transcriptome analysis and differential expression analysis

Global mRNA-expression were determined using SurePrint G3 Human Gene Expression 8x60k V2 (AMADID 39494) microarrays (Agilent Technologies, Santa Clara, USA) according to the manufacturer’s protocol. After hybridization (17 h, 65 °C) the arrays were scanned with a G2505C Sure Scan Microarray Scanner, raw data were extracted as text files using the Feature Extraction 10.7 software (Agilent Technologies), and imported into the R statistical platform [30]. Two technical replicates were generated for the CLIP2 overexpressing clones and two technical plus two biological replicates were generated for the siRNA knockdown experiments. The expression data from technical replicates were averaged. Data quality assessment, pre-processing, normalization (quantile normalization), and data analyses were carried out with the statistical software R using the *limma* package available from bioconductor [31, 32]. Differential expression analysis was performed between the normal CLIP2-expressing- and the three CLIP2-overexpressing TPC-1 clones, each, using the linear model component within *limma*. A minimum log2-fold change of 0.5 and a significance level of adjusted *p*-values FDR < 0.1 after Benjamini-Hochberg correction were applied to identify differentially expressed genes for each comparison. Finally, the consensus between genes found to be differentially expressed in each of the three comparisons was identified.

### Pre-selection of genes for GAN reconstruction

**Fig. 1:**
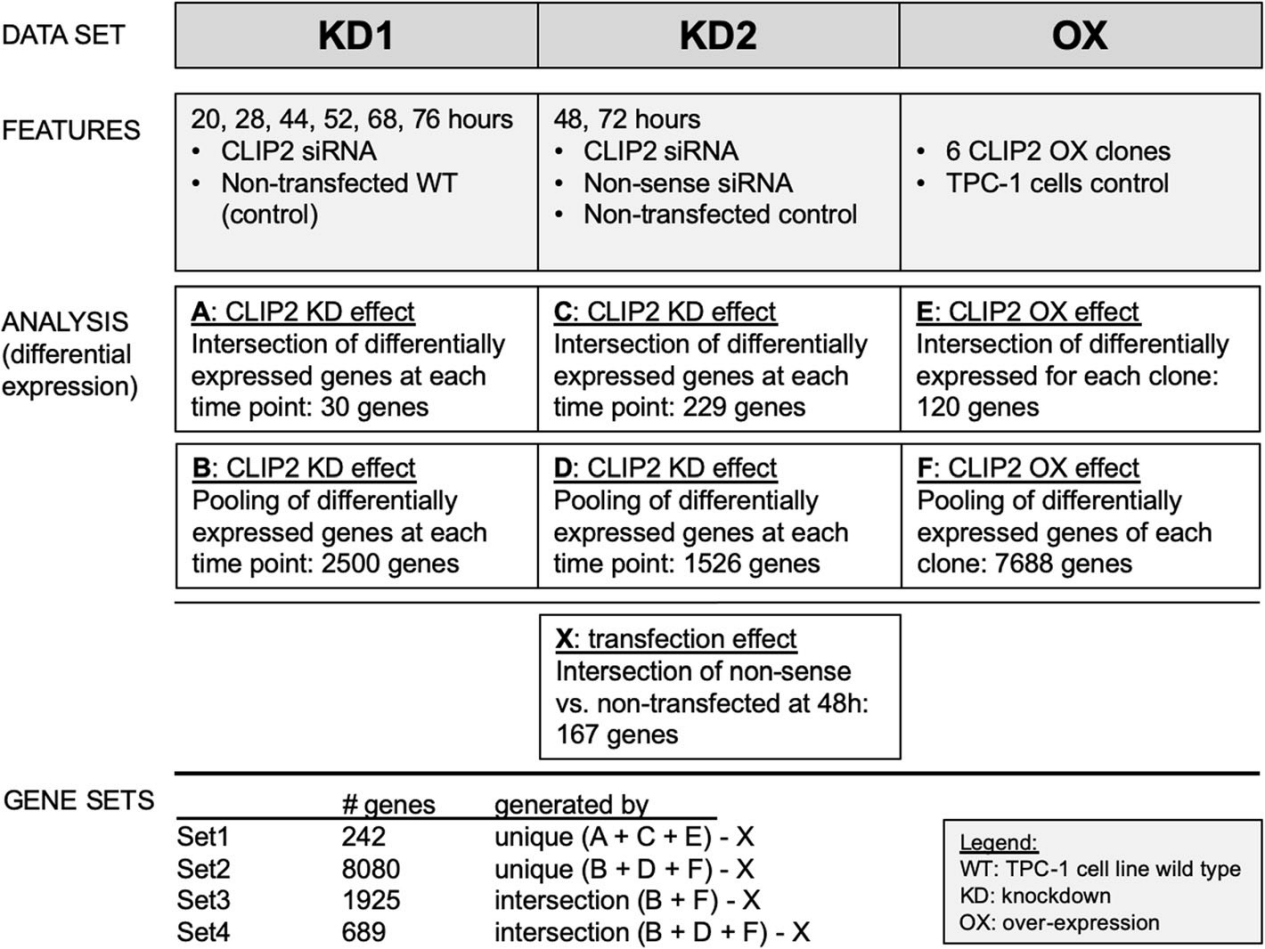
Gene set generation based on CLIP2 knockdown and CLIP2 overexpression. Four different gene sets were generated based on differential gene expressions in the three data sets and there perturbed CLIP2 levels by siRNA knockdown and stabile CLIP2 overexpression in TPC-1 cells

The pre-selection of the gene sets which were subjected to network reconstruction was based on differential gene expression between wild type TPC-1 cells (WT) and CLIP2-perturbed TPC-1 cells by CLIP2 siRNA knockdown or stabile CLIP2 overexpression, respectively. Figure 1 gives an overview of the differentially expressed gene sets and the generation of four different gene sets for the GAN reconstruction.

### GAN reconstruction with the GeneNet method

Preselected sets of genes were subjected to gene association network reconstruction using the regularized dynamic partial correlation method implemented in the *GeneNet* R-package [33]. Gene expression data were presented as continuous log2-transformed and quantile normalized array-signal-intensities that approximate a normal distribution and are therefore suitable for partial correlation analysis. Pairwise relationships between genes were inferred based on a dynamic Bayesian network model with shrinkage estimation of covariance matrices as implemented in the *GeneNet* R-package available from CRAN [34]. Edge directions were not considered. In order to assess the complexity of the resulting networks, the density of each network was compared to the density of the Reactome functional interaction network [35, 36]. In order to get to an optimal edge probability cut-off, which is an important parameter in the *GeneNet* network reconstruction process, values between 0 and 0.95 (0.05 steps) in addition to 0.96, 0.97, 0.98, 0.99, 0.999 were used in *GeneNet* GAN reconstruction for each gene set to be compared to the reference networks.

### Evaluation of *de-novo* reconstructed networks

#### Assessment of plausibility

In order to assess the plausibility of the de novo reconstructed gene association networks (GANs), we compared the presence of any interaction with that of those in the Reactome (https://reactome.org), Biogrid (https://thebiogrid.org), and STRING (https://string-db.org) database networks. The publicly available networks are based on literature research and experimentally validated interactions and mostly represent protein-protein interactions. After download, sub-networks from the reconstructed and the database networks were built that exclusively contained genes overlapping between the reconstructed and the publicly available interaction networks. The number of common edges was determined and related to the total number of edges in the GAN (1st-order comparison). Since GANs are based on correlation between two genes which in reality can reflect the interplay of several proteins we allowed for a 2nd and 3rd-order comparison for which edges between all second neighbors and/or third neighbors were added to the publicly available networks. Permutation testing was performed to assess if the number/percentage of common edges in the reconstructed network was significantly higher than in randomized networks composed of the same genes. Random networks were generated by permutation of the node names in the network, while preserving the topology of the reconstructed network. For each permutation (*n* = 1000) the number/percentage of common edges with the reference sub-networks was determined. The reconstructed network was considered significantly better than random, if more than 90% of the random sub-networks contained lower numbers/percentages of common edges with the respective database network than the reconstructed network (*p*-value < 0.1).

#### Shortest-path analysis

Additionally, a mean shortest path concept was used for the evaluation of the reconstructed networks [37, 38]. The shortest paths in the reference network of all proposed direct interactions in the reconstructed network were averaged (mean shortest path) and compared to the mean shortest path after permutation of the reconstructed network (as described above). The reconstructed network was considered non-random, when 90% of the random networks generated by permutation showed a greater mean shortest path length. Functions and data structures from the *igraph* R package were used [37].

### Network analysis

Graph topology analyses based on centrality measures were applied in order to determine the importance of each node in the reconstructed association networks [37, 38]. The three centrality measures *degree*, *shortest path betweenness* and *closeness* [39] were combined into one consensus centrality measure: For each node the three centrality values where ranked followed by building the mean. The consensus centrality measure is intended to identify nodes that are central but also directly connected to other central nodes and therefore are “important” within the network. Additionally, a pathway enrichment analysis using the genes within the 1st- and 2nd-neighborhod of CLIP2 within the prioritized reconstructed network (gene set 4, edge probability cut-off 0.5) and the Hallmark gene set collection from the MSIG database (https://www.gsea-msigdb.org/gsea/msigdb/index.jsp) was carried out. Fisher’s exact test followed by Benjamini & Hochberg multiple-testing correction of resulting *p*-values was performed. Gene sets with adjusted *p*-values less than 0.05 were considered as statistically significantly enriched [40].

## Results

### siRNA knockdown of CLIP2 mRNA and protein expression

**Fig. 2:**
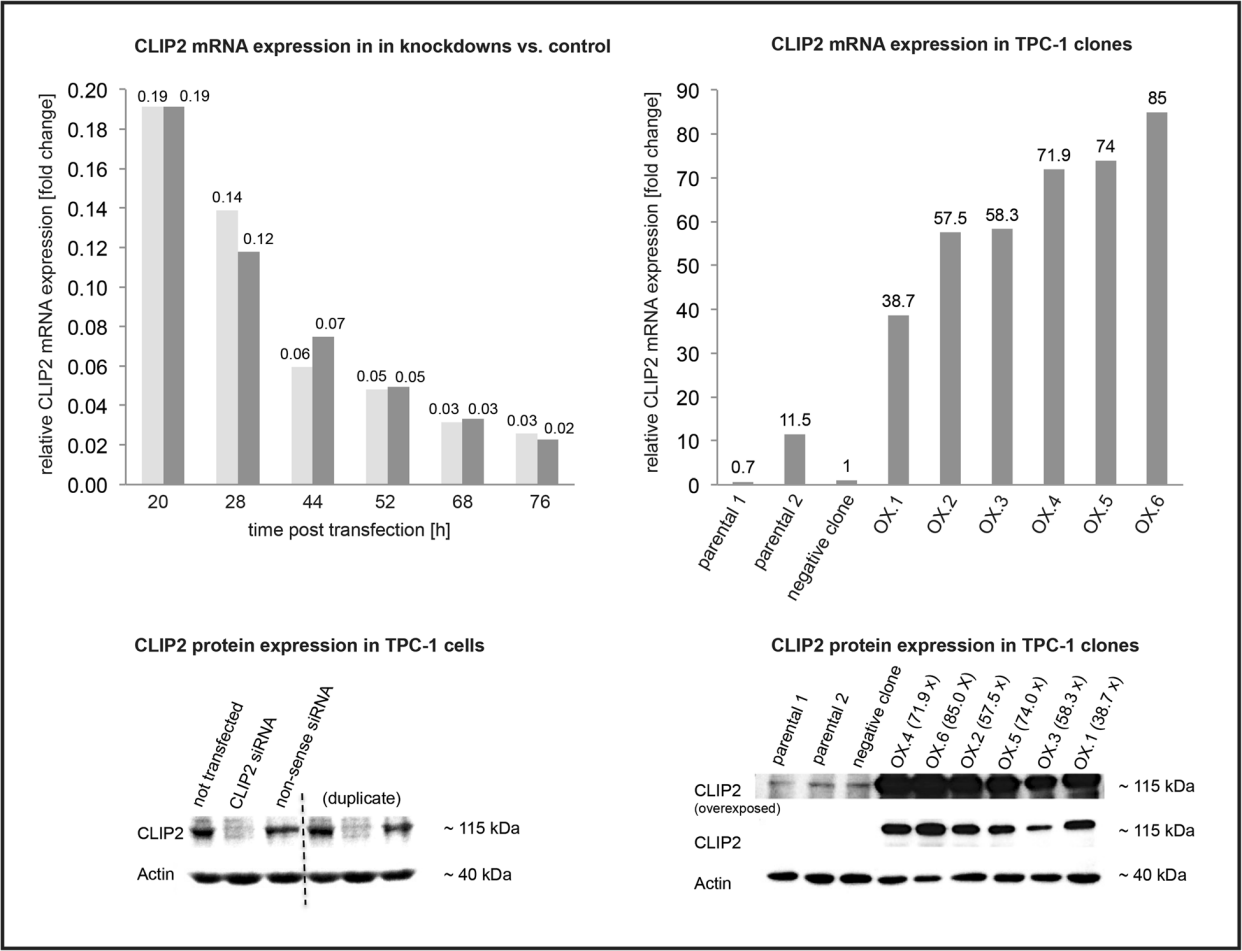
CLIP2 mRNA and protein expression. Top left: mRNA expression in CLIP2 siRNA knockdown TPC-1 cells compared to non-transfected control (duplicates, two bars per time point). top right: mRNA expression in CLIP2 overexpressing TPC-1 cell clones compared to negative clone (relative expression). bottom left: CLIP2 and Actin protein expression in non-transfected TPC-1 cells, CLIP2 siRNA transfected TPC-1 cells (knockdown), and non-sense transfected TPC-1 cells. Bottom right: CLIP2 and Actin protein expression in TPC-1 clones with CLIP2 overexpression, negative clone (no CLIP2 overexpression), and TPC-1 parental cells

The CLIP2 mRNA levels over time after the CLIP2 knockdown by RNAi are visualized in Fig. 2. The minimum CLIP2 mRNA level was observed at 76 h with approximately 2.5% of the mRNA level compared to the non-transfected, identically treated control TPC-1 cells after 76 h. Compared to the untreated TPC-1 cells at timepoint t_0_, the minimal CLIP2 mRNA level was reached at 52 h with approximately 10% of the initial CLIP2 mRNA level. The CLIP2 Protein knockdown was visualized by Western Blot and shows a substantial reduction of the CLIP2 protein level in CLIP2 siRNA transfected cells compared to non-treated and non-sense siRNA transfected cells and thereby proves the efficiency of the siRNA knockdown.

### CLIP2 mRNA and protein overexpression in TPC-1 clones

By lentiviral transduction, six different TPC-1 clones were generated (named OX.1 – OX.6) exhibiting 38-fold, 57-fold, 58-fold, 72-fold, 74-fold, and 85-fold CLIP2 mRNA overexpression compared to the parental TPC-1 cells and a transduction-negative TPC-1 cell clone, respectively (Fig. 2). Western blot analysis revealed a broad range of CLIP2 protein levels in the different clones, which are in line with corresponding mRNA levels (Fig. 2).

### Gene sets for network reconstruction

Four sets of differentially expressed genes were determined to be associated with the CLIP2 perturbation and were generated following the rationales illustrated in Fig. 1. A set of 167 genes was determined to be differentially expressed due to the transfection procedure and contains genes that were differentially expressed between CLIP2 siRNA and non-treated cells, as well as between non-sense siRNA transfected and non-treated cell 48 h post transfection. These 167 genes were excluded from the gene sets used for the network reconstructions. The final four gene sets (set 1 – set 4) comprise 242, 8080, 1925, and 689 genes, respectively. A venn diagram and an upset plot visualize the intersections between the gene set in SI Figure 1.

### Evaluation of reconstructed networks and prioritization of one reconstructed network

**Fig. 3:**
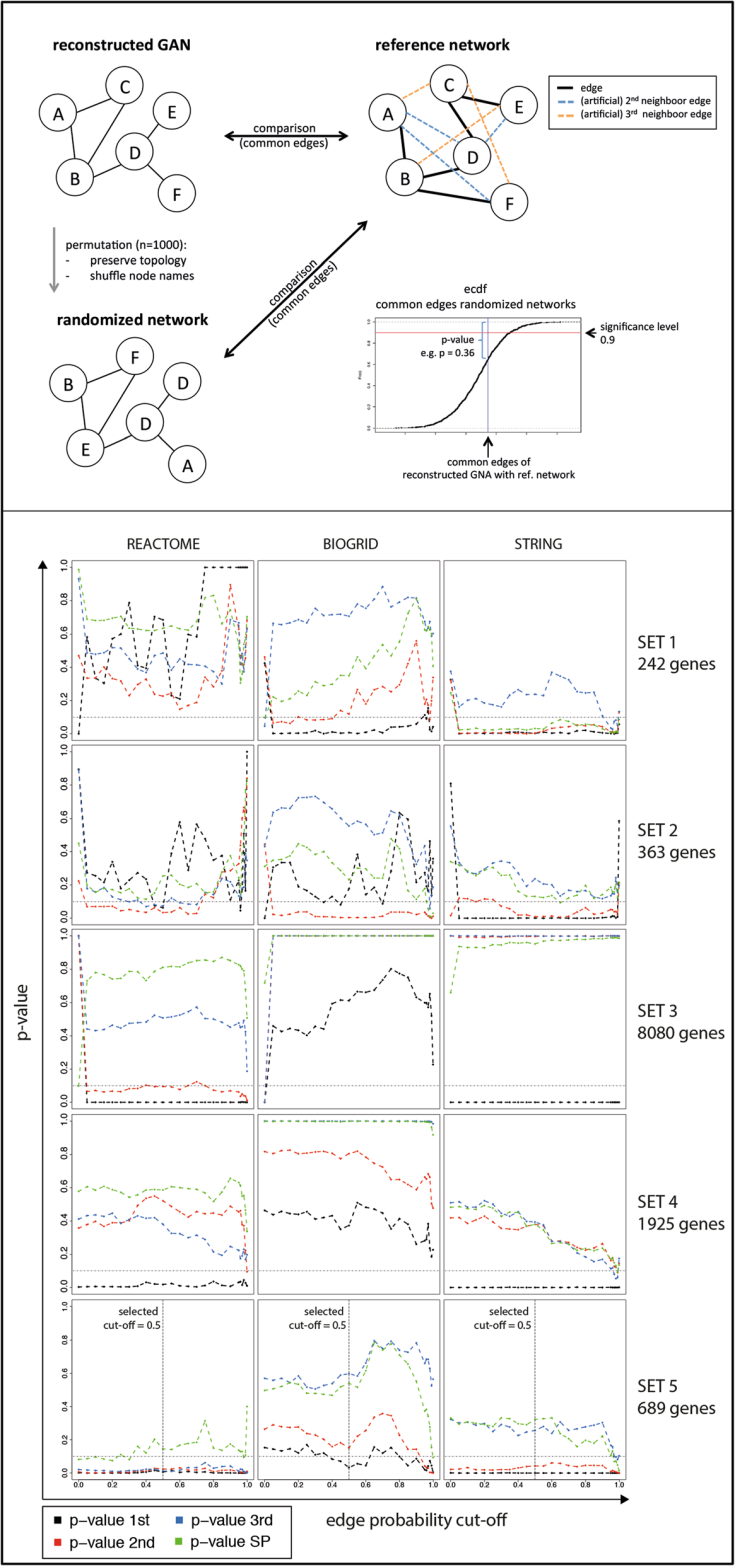
Network evaluation. Upper panel: Concept of permutation test for network evaluation statistics. Lower panel: rows correspond to analyzed gene sets and columns to the data base networks. *P*-values are permutation derived and indicate the percentages of random networks that performed better than the reconstructed network for each edge probability cut-off in the *GeneNet* network reconstruction process

Network evaluation is based on the concept introduced in the Material and Methods section, were the reconstructed networks are compared to existing reference networks from the Reactome, Biogrid, and String database. The quantitative metric to compare the validity and plausibility of a network we used is the *p*-value derived from the permutation approach and subsequent identification of common edges with the reference network (Fig. 3, top panel).

When comparing the Reactome network with the GAN 1st-, 2nd-, and 3rd order edges, significantly more common edges resulted from the GANs reconstructed from gene set 4 compared to randomly generated networks with identical topology (RNIT) but permutated node names (*p* < 0.1), regardless of the chosen edge-probability-cut-off (Fig. 3 bottom panel, left column: comparison to Reactome network). The networks reconstructed from gene set 4 exhibit *p* < 0.1 for all comparisons and cut-off values, except the shortest-path comparisons were no significant results were obtained. Also, with regard to mean shortest paths, gene set 4 resulted in the lowest *p*-values amongst the compared gene sets. When using the Biogrid interaction network as reference, again gene set 4 showed superior results with regard to common edges and shortest path analysis (Fig. 3, bottom panel). In the comparison with the STRING interaction network, gene sets 1 and 4 yielded comparable results (permutation *p*-values) with regard to common edges with the GANs and shortest paths, while gene sets 2 and 4 showed inferior overlap compared to the others. Overall, gene set 4 with 689 genes resulted in GANs with the best overall performance (Fig. 3, bottom panel). For the final network, gene set 4 with an edge-probability-cut-off of 0.5 was chosen. For two reasons: Firstly, only edges with a probability of greater than 50% were considered, in order to obtain a network consisting of edges with greater likelihood than random edge assignment. Secondly, the network includes CLIP2 with 20 direct interaction partners, which is a feasibly number of genes for further wet-lab studies. Greater edge-probability-cut-offs result in networks with very few interaction partners of CLIP2 or networks not containing CLIP2 and are hence not useful to derive novel knowledge on the biological function of CLIP2 and the identification of CLIP2 interaction partners.

### Network description and network analysis

**Fig. 4:**
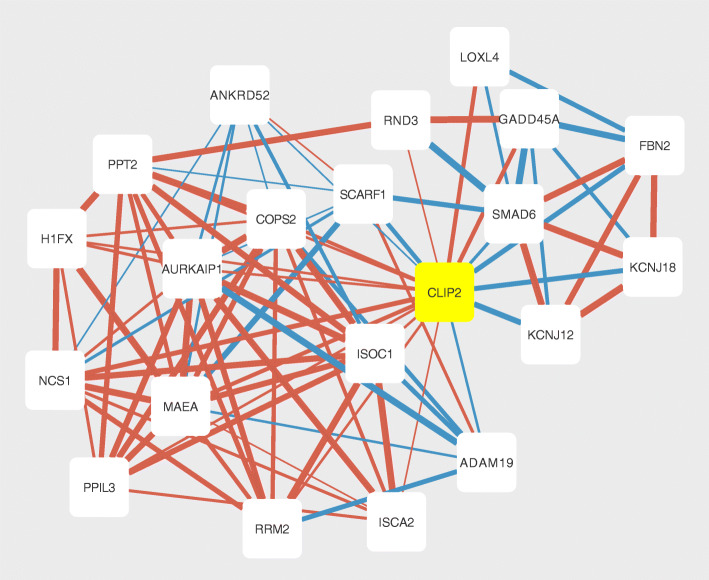
CLIP2 1st-neighbourhood gene association network. CLIP2 centered 1st-neighbourhood network generated with gene set 4 (see Fig. 1) and an edge-probability cut-off of 0.5 in the *GeneNet* network reconstruction. Blue colored edges indicate positive correlations, red colored edges indicate negative correlations. The line width of the edges corresponds to the edge-probability derived from the *GeneNet* method

The final GAN was reconstructed from gene set 4 using an edge-probability cut-off of 0.5, which resulted in a network with 654 nodes and 10,777 edges (SI Table 3). The number of reconstructed direct CLIP2 interactors, dependent on the selected probability cut-off during network reconstruction and the degree distribution of the selected network (gene set 4, cut-off > 0.5, degree distribution follows power law), are visualized in SI Figure 2. The first neighborhood of the CLIP2 gene in this network consisted of the genes KCNJ18, PPT2, H1FX, RND3, NCS1, KCNJ12, ADAM19, SCARF1, GADD45A, PPIL3, SMAD6, ISOC1, ANKRD52, RRM2, AURKAIP1, LOXL4, MAEA, ISCA2, FBN2, COPS2 (Fig. 4). According to the consensus centrality measure, six nodes of the 1st neighborhood of CLIP2 were among the 100 most important genes of the entire network: PPT2 (rank 20), SMAD6 (rank 64), RND3 (rank 78), KCNJ12 (rank 89), NCS1 (rank 93), and SCARF1 (rank 99). From the 100 most important genes in the whole GAN, 98 were part of the CLIP2 2nd neighborhood (total 363 genes, SI Table 4). Pathway enrichment analysis with the 1st and 2nd-neighborhod genes (*n* = 363, SI Table 5) and the Hallmark gene set collections revealed a significant enrichment with adj-*p*-values < 0.05 in 19 out of 50 gene sets. The results are summarized and visualized in SI Figure 3 and the statistical results are in SI Table 6. The top five enriched gene sets include HALLMARK_TNFA_SIGNALLING_VIA_NFKB, HALLMARK_KRAS_SIGNALING_UP, HALLMARK_HYPOXIA, HALLMARK_P53_PATHWAY, and HALLMARK_MESENCHYMAL_TRANSITION.

### qRT-PCR validation of reconstructed network

Out of the 20 genes that built the first neighborhood of CLIP2 in the reconstructed network, ADAM19, ISCA2, ISOC1, MAEA, PPIL3, and RRM2 exhibited a significant correlation test result (FDR < 0.1) when comparing their expression with that of CLIP2 after qRT-PCR (SI Table 7).

### Protein co-expression of CLIP2 and PPIL3 by IHC

Immunohistochemical staining of consecutive FFPE tumor sections from PTC patients demonstrated co-expression of the CLIP2 and PPIL3 proteins (SI Figure 4). The observed staining patterns for CLIP2 and PPIL3, respectively, were well comparable with respect to localization and staining intensities.

## Discussion

This study presents the gene association network of CLIP2, which has been previously associated with radiation induced papillary thyroid carcinoma (PTC) in young patients [22]. We further developed a PTC radiation marker by combining CLIP2 protein expression with CLIP2 gene copy number status, for which we found a positive dose-relationship in children exposed to ionizing radiation at ages below 5 years [23]. In the course of therapeutic irradiation of the head and neck region, the thyroid gland often receives doses of ionizing radiation comparable to those after the Chernobyl reactor accident. This implies a potential clinical relevance of the CLIP2 radiation marker, while its functional role in radiation-induced papillary thyroid carcinoma is of interest [41, 42]. Although PTC generally has a good prognosis, about 10–30% of the PTC become recurrent, which is associated with a much worse prognosis. This motivates a deeper functional understanding of PTCs for possible improvements in therapy [43]. A first functional association of CLIP2 with cancer-related processes such as genomic instability, mitogen-activated protein kinase signaling, and apoptosis, was derived from de novo a network reconstructed network from PTC samples from radiation exposed patients [44]. In this study, we aimed to elucidate the functional role of CLIP2 by *GAN* reconstruction of CLIP2 perturbed PTC in vitro models with the aim to identify the potential interaction partners of CLIP2 for further investigation.

One major task in GAN reconstruction is the pre-selection of a set of genes for the sake of reducing complexity and noise. As no consent about the approach exists, most often, intuitively generated assumptions such as selecting genes according to the level of statistical significance or variance across samples are used. Further, different GAN reconstruction approaches exist, all of which resulting in probability based networks requiring experimental validation [45]. We generated two CLIP2 perturbation data sets with the CLIP2 gene transiently down-regulated by siRNA and one with stable upregulated CLIP2 expression. For GAN reconstruction gene preselection, we combined the significantly differentially expressed genes between cells with up- and downregulated CLIP2 in four different ways. The reconstruction of GANs was subsequently run on these gene sets with a series of different edge probability cutoffs.

The resulting GANs were subjected to validation by comparing them to curated human interaction networks (interaction-database network, DBN) as provided by Reactome, Biogrid and STRING and defined the percentage of common interactions as evaluation measure. Further, we tested whether the overlap of the GANs with DBNs was better than with randomly generated networks. Generally, we expect an interaction of two genes/proteins present, to be also present in the reconstructed network. In order to consider for the fact that gene-associations can reflect direct, but also indirect protein-protein interactions, for the comparisons in the GANs we allowed additional edges from the second and third neighborhoods. The best performing GAN in terms of overlap with the three DBNs was generated from the gene set in which the intersection between all genes that were differently expressed between cells with CLIP2-siRNA down-regulation after 22, 28, 44, 52, 68 and 76 h and transfection control and that differently expressed between TPC-1 cells overexpressing and non-overexpressing cells.

The GAN contained 20 genes in the first neighborhood of CLIP2. The genes GADD45A, COPS2, AURKIP1, and ANKRD52 are associated with DNA repair/maintenance after stressful growth arrest after DNA damage as introduced by e.g. ionizing radiation. GADD45A directly interacts with AURKA (Aurora-A) and is involved in in the regulation of DNA repair, cell cycle, cell proliferation, and apoptosis [46]. Additionally, GADD45A is part of the mitogen activated kinase (MAPK) pathway where it binds to MTK1 and thereby enables an interaction with MKK6 [47]. COPS2 is an essential subunit of the multiprotein complex constitutive photomorphogenesis 9 signalosome (COP9) that is involved in the phosphorylation of p53/TP53, JUN, and NFKBIA, with an important role in processes such as DNA double strand repair (DSR) and thereby plays a role in the suppression of apoptosis and the response to ionizing radiation [48–50]. The AURKA interacting protein (AURKIP1) was reported to initialize a proteasome-dependent, but ubiquitin-independent degradation of AURKA [51]. AURKA is, similar to COSP2/COP9, involved in the phosphorylation of p53/TP53, particularly the checkpoint-response pathways critical for the oncogenic transformation of cells [52]. It is also known that mitotic AURKA plays a major role in the spindle positioning and organization of the microtubule during mitosis [53]. AURKA overexpression leads to aneuploidy, chromosomal instability and cellular transformation and is considered to be an oncogene [51, 54, 55]. Therefore, a deregulation of the AURKIP1 initialized degradation of AURKA by the overexpression of CLIP2 could potentially initialize the carcinogenesis of PTC after radiation exposure. Additionally, the conducted pathway enrichment analysis on the 1st- and 2nd-neighborhod genes of CLIP2 suggest a functional involvement of CLIP2 in the thyroid carcinogenesis. The most prominent findings were their involvement in TNFA via NFKB signaling, an upregulated KRAS signaling, in P53 and apoptosis pathways, along with interactions with immunologically and cancer relevant mechanisms such as E2F-target, IL2-STAT5 signaling, inflammatory response. Moreover, the involvement of CLIP2 and it’s 1st + 2nd neighborhood genes in early-state carcinogenic process of chromosomal instability was show via the enrichment of Hallmark gene sets G2M-checkpoint and mitotic spindle, which suggest an involvement in chromosomal segregation. This finding integrates well with previous functional associations of CLIP2 with chromosomal instability, derived from a GNA that was generated from transcriptomic data on PTC samples from patients exposed to ionizing radiation [44]. The network is visualized in SI Figure 5. Interestingly, the PPIL3 gene is also in the first neighborhood of the GAN generated from patient PTC samples underlining the possible radiation-associated role of the interaction [22]. PPIL3 belongs to the cyclophilin (CyP) family [56] which act as molecular catalysts or chaperones in protein folding, protein assembly, or repair and are functionally associated with mitochondrial maintenance, apoptosis, cell cycle progression, T-cell regulation, and inflammation, all of which cancer-related processes [56–61]. The validation of the CLIP2-PPIL3 interaction at mRNA and protein level makes PPIL3 a very interesting candidate for further investigations in wet lab studies. ANKRD52 is a subunit of the PP6 multi-subunit enzyme and might play a role in PP6 activity during S-phase [62].

LOXL4, ADAM19, FBN2, and RND3 are in the second group with close functional similarities and are involved in extracellular matrix interactions. LOXL4 encodes a copper-dependent amine oxidase that is involved in the formation of crosslinks in collagens and elastin and is a potential drug target for the treatment of head and neck squamous cell carcinoma, where it is differentially expressed between tumor and normal tissue [63, 64]. LOXL4 is also known to promote proliferation and metastasis of gastric cancers and breast cancer [65, 66]. ADAM19 is a member of the ADAM family (a disintegrin and metaloprotease domain) and is known to be involved in fertilization, muscle development, and neurogenesis but also in cell-cell and cell-matrix interactions. ADAM19 was also reported as a miRNA target in the treatment of several cancers such as colorectal cancer, non-small cell lung cancer, and retinoblastoma, with the goal to inhibit proliferation, migration and invasion [67–69]. The FBN2 protein is part of the connective tissue microfibrils and involved in the degradation of the extracellular matrix. FBN2 plays a role in the ERK signaling pathway and its DNA hypermethylation was recently discussed as a biomarker for colorectal cancer [70, 71]. RND3 is a member of the GTPase protein superfamily, is thereby also linked to the ERK signaling pathway and is known to contribute to the disassembly of actin filaments [72, 73]. Another study reports an under-expression of RND3 (RohE) in several cancer types compared to the corresponding normal tissue and suggests a tumor suppressing effect through p53-activation [74]. Thus, the genes LOXL3, ADAM19, FBN2, and RND3 are associated with cancer relevant biological processes such as loss of adhesion, proliferation, migration and metastasis and are therefore might be functionally related to CLIP2 in the context of radiation induced PTC-carcinogenesis of PTC. Apart from the above mentioned direct CLIP2 interaction partner PPIL3 in the GNA reconstructed from papillary thyroid carcinoma patient data, no common direct interactions within the herein reconstructed GNA and the STRING and Biogrid networks were observed (Fig. 4, SI Figure 5). One potential reason may be the very specific focus of this study, which was elucidation of the functional role of CLIP2 in radiation associated thyroid carcinoma, while the interactions in the STRING and Biogrid database originate from various tissue types cell culture models. A potential limitation of this study could be the limited sample size available for network reconstruction (*n* = 30), which was recommended in a previous study for GNA reconstruction between 100 and 500 samples to obtain robust networks [75]. Since the expression data generated within this study were specifically generated from CLIP2 perturbed cell lines, with great dynamic range of CLIP2 expressions, the limitation in sample size may be partly compensated. However, the functional context of CLIP2 in the database-derived networks, and the GANs reconstructed from clinical PTC-samples and the herein used cell culture model is identical with respect to the role of CLIP2 in chromosomal segregation, the transport along the microtubule, and hence, cell cycle and proliferation.

This study presents a functional characterization of the CLIP2 gene derived from a GAN from the thyroid cancer cell line TPC-1 after perturbation of the CLIP2 mRNA expression. Additionally, we suggest an a priori knowledge based approach for the evaluation of reconstructed gene association networks through comparison with existing database networks. The derived functional context of CLIP2 with respect to the development of PTC integrates well with our previous functional characterization of CLIP2 in clinical samples of PTC patients and published knowledge on the proposed direct interaction partners of CLIP2 in the herein presented gene association network. Literature knowledge on the direct interaction partners strongly suggests an association of CLIP2 with cancer relevant processes such as DNA repair, chromosomal instability and promoted proliferation.

These findings further support the potential of CLIP2 as a marker for radiation induced PTCs in young patients as previously suggested [22, 23, 76, 77]. Most remarkable, the PPIL3 gene was found to be a direct CLIP2 interaction partner in the present study and confirmed the finding in data generated from clinical PTC samples. Additionally, protein co-expression of PPIL3 and CLIP2 clinical radiation-associated PTC samples supports the existence of CLIP2-PPIL3 interaction.

In our study we identified the gene association network of the radiation marker CLIP2 and thereby provide new insights into the functional role of the gene in radiation-associated thyroid carcinogenesis. We applied a novel knowledge-based optimization workflow for gene association network reconstruction.

## Supplementary information

**Additional file 1: SI Figure 1.** Comparison of the four gene sets used for GNA reconstruction, generated by differential expression analysis (SI Figure 1) A) Upset plot in intersect mode (common genes within the respective gene sets green: present in all four gene sets, black: present in three gene sets, blue: present in two gene sets, orange: number of genes in gene sets 1-4) B) Upset plot in distinct mode (common genes within the respective gene sets, but unique to the respective gene sets / not present in other gene sets) C) Venn diagram D: Visualization of GAN reconstruction statistics derived from GeneNet method applied to gene set 4. Each dot in the plot represents a potential edge between two genes in the network. A total number 237016 edges was calculated from gene set 4, while the application of the edge probability cut-off of 0.5 (black dashed line) results in the final network with 10777 edges and 654 nodes (genes). All direct interactions with CLIP2 (1st neighborhood) are visualized. Negative partial correlation coefficients represent a negative association, while positive partial correlation coefficients indicate a positive association between the genes (nodes).

**Additional file 2: SI Figure 2.** A) number of direct CLIP2 interactors in the GNA reconstructed from gene set 4, dependent on the edge probability cut-off in the GeneNet method. Red dashed line indicates the selected cut-off of 0.5 B) Edge-list of the 1st neighborhood of CLIP2 reconstructed from gene set 4. pcor = partial correlation coefficient, node 1/2 = gene names of nodes, pval = *p*-value of partial correlation test, qval = q-values (positive false discovery rate), prob = edge probability black line: selected cut-off of 0.5 C) Degree distribution of the GNA generated from gene set 4 and a probability cut-off of 0.5 in the GeneNet reconstruction method. The degree distribution (approximately) follows a power law and thereby represents on attribute of a scale-free network.

**Additional file 3: SI Figure 3.** Pathway enrichment analysis using the 50 Hallmark gene sets form MSigDB Fischer Exact Test was applied to test for enrichment of Hallmark gene sets within the 2nd-neighborhood of CLIP2 of the GNA reconstructed from gene set 4. 19 out of 50 gene set exhibited a significant enrichment with an adjusted *p*-value < 0.05 (red dashed line)

**Additional file 4: SI Figure 4.** Validation of CLIP2 and PPIL3 protein co-expression by Immunohistochemical staining in papillary thyroid tumor tissue (10 cases) left column: CLIP2 staining right column: PPIL3 staining

**Additional file 5: SI Figure 5.** First neighborhood networks of CLIP2 A) CLIP2 first neighborhood network extracted from BIOGRID database. Interactions of genes/proteins with a blue background color were derived from human biological material, while interactions with genes with a yellow background were derived from mouse biological material. B) CLIP2 first neighborhood network extracted from STRING database. Only interactions based of experimental data or co-expression are visualized. C) CLIP2 first neighborhood network reconstructed within this study D) CLIP2 first neighborhood network reconstructed from human patient gene expression data (papillary thyroid carcinoma) of the post-Chernobyl UkrAm cohort.

**Additional file 6: SI Table 1.** STR typing TPC-1 parental and clones

**Additional file 7: SI Table 2.** TagMan probes for qRT-PCR validation of 1st-neigboors of CLIP2

**Additional file 8: SI Table 3.** Edgelist for GNA from gene set 4 and probability cut-off > 0.5

**Additional file 9: SI Table 4.** Ranking of network genes according to summarized centrality measure (see MM)

**Additional file 10: SI Table 5.** 1st and 2nd neighborhood genes of CLIP2 in GNA from gene set 4 and edge-probability cut-off > 0.5

**Additional file 11: SI Table 6.** Pathway enrichment analysis based on hypergeometrical test and HALLMARK gene sets

**Additional file 12: SI Table 7.** Correlation test of 1st-neighboors of CLIP2 using qRT-PCR data

## Data Availability

The datasets used and/or analyzed during the current study are available from the corresponding author on reasonable request.
