# Supplementary material for: Transcriptome network of the papillary thyroid carcinoma radiation marker CLIP2
Source: Radiat Oncol. 2020 Jul 29;15:182. doi: 10.1186/s13014-020-01620-5 (PMC7392692; doi:10.1186/s13014-020-01620-5)
Supplement: Supplementary file 1 — Additional file 1: SI Figure 1. Comparison of the four gene sets used for GNA reconstruction, generated by differential expression analysis (SI Figure 1) A) Upset plot in intersect mode (common genes within the respective gene sets green: present in all four gene sets, black: present in three gene sets, blue: present in two gene sets, orange: number of genes in gene sets 1-4) B) Upset plot in distinct mode (common genes within the respective gene sets, but unique to the respective gene sets / not present in other gene sets) C) Venn diagram D: Visualization of GAN reconstruction statistics derived from GeneNet method applied to gene set 4. Each dot in the plot represents a potential edge between two genes in the network. A total number 237016 edges was calculated from gene set 4, while the application of the edge probability cut-off of 0.5 (black dashed line) results in the final network with 10777 edges and 654 nodes (genes). All direct interactions with CLIP2 (1st neighborhood) are visualized. Negative partial correlation coefficients represent a negative association, while positive partial correlation coefficients indicate a positive association between the genes (nodes). [file 13014_2020_1620_MOESM1_ESM.pdf]

SI Figure 1

A)

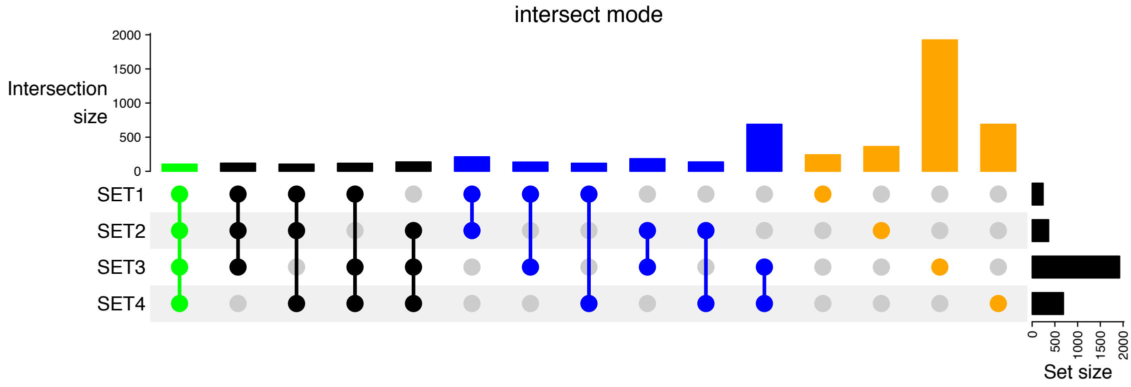

B)

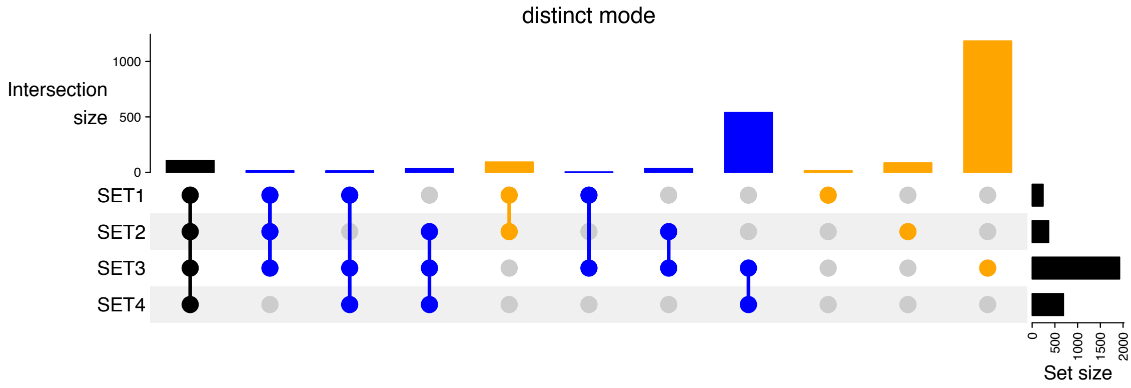

C)

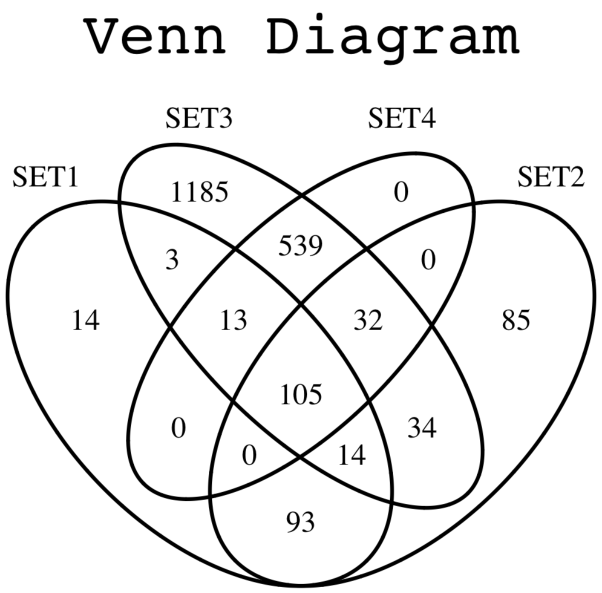

SI Figure 1: Comparison of the four gene sets used for GNA reconstruction, generated by differential expression analysis (SI Figure 1)

A) Upset plot in intersect mode (common genes within the respective gene sets green: present in all four gene sets, black: present in three gene sets, blue: present in two gene sets, orange: number of genes in gene sets 1-4)
B) Upset plot in distinct mode (common genes within the respective gene sets, but unique to the respective gene sets / not present in other gene sets)
C) Venn diagram

SI Figure 1: D)

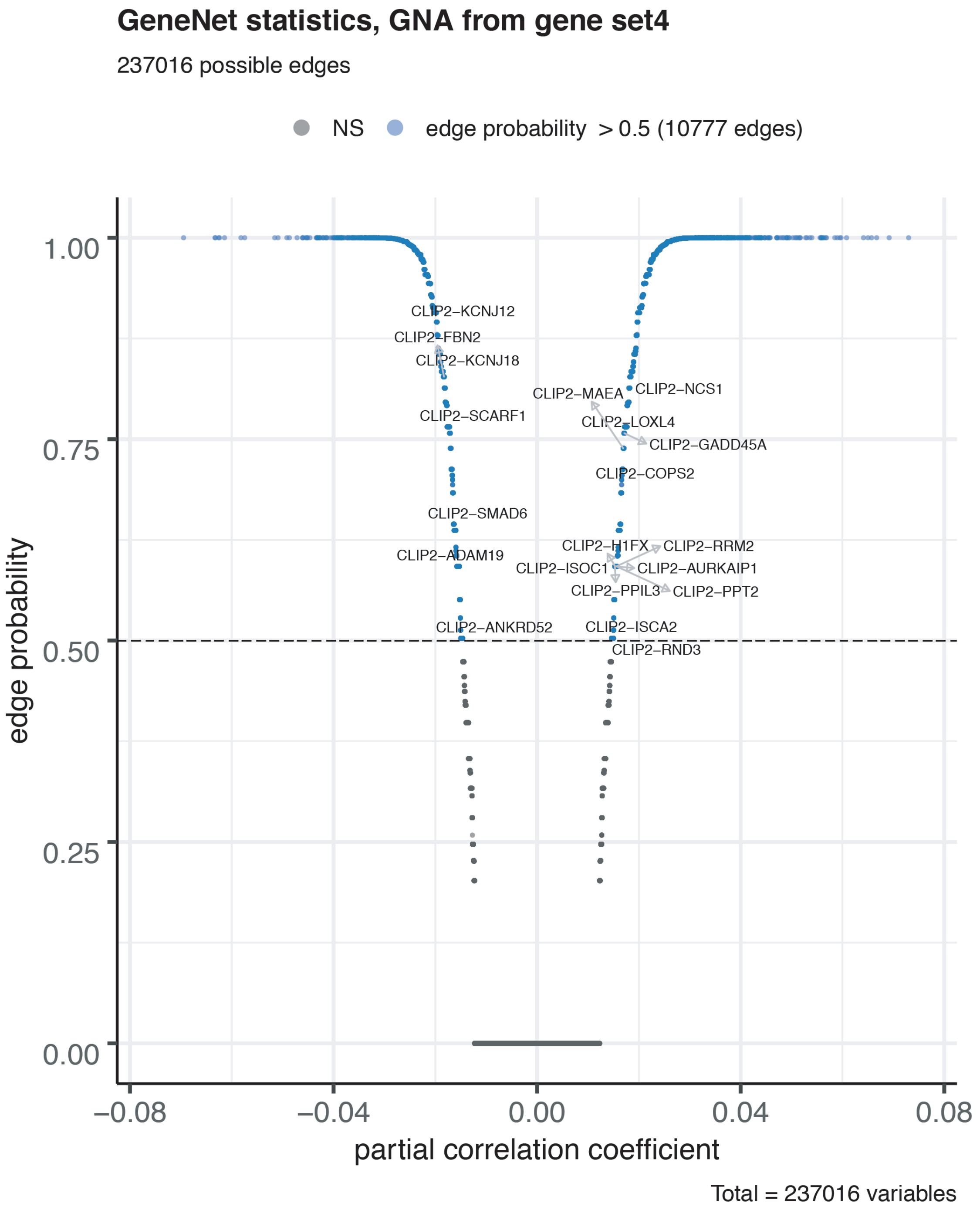

SI Figure 1: D: Visualization of GAN reconstruction statistics derived from GeneNet method applied to gene set 4. Each dot in the plot represents a potential edge between two genes in the network. A total number 237016 edges was calculated from gene set 4, while the application of the edge probability cut-off of 0.5 (black dashed line) results in the final network with 10777 edges and 654 nodes (genes). All direct interactions with CLIP2 (1st neighborhood) are visualized. Negative partial correlation coefficients represent a negative association, while positive partial correlation coefficients indicate a positive association between the genes (nodes).
