# Supplementary material for: Transcriptome network of the papillary thyroid carcinoma radiation marker CLIP2
Source: Radiat Oncol. 2020 Jul 29;15:182. doi: 10.1186/s13014-020-01620-5 (PMC7392692; doi:10.1186/s13014-020-01620-5)
Supplement: Supplementary file 2 — Additional file 2: SI Figure 2. A) number of direct CLIP2 interactors in the GNA reconstructed from gene set 4, dependent on the edge probability cut-off in the GeneNet method. Red dashed line indicates the selected cut-off of 0.5 B) Edge-list of the 1st neighborhood of CLIP2 reconstructed from gene set 4. pcor = partial correlation coefficient, node 1/2 = gene names of nodes, pval = p-value of partial correlation test, qval = q-values (positive false discovery rate), prob = edge probability black line: selected cut-off of 0.5 C) Degree distribution of the GNA generated from gene set 4 and a probability cut-off of 0.5 in the GeneNet reconstruction method. The degree distribution (approximately) follows a power law and thereby represents on attribute of a scale-free network. [file 13014_2020_1620_MOESM2_ESM.pdf]

SI Figure 2

A)

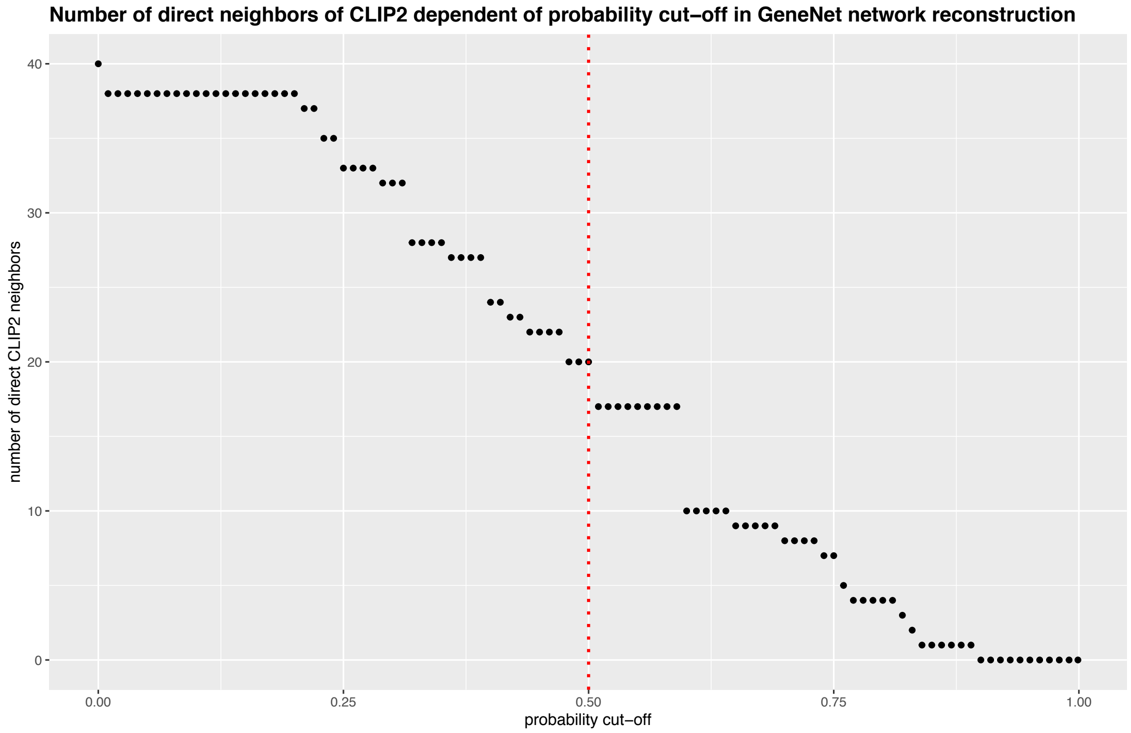

B)

pcor	node1	node2	pval	qval	prob
-0.0197	CLIP2	KCNJ12	0.0006	0.0322	0.8954
-0.0188	CLIP2	KCNJ18	0.0010	0.0485	0.8339
-0.0183	CLIP2	FBN2	0.0013	0.0595	0.8274
0.0181	CLIP2	NCS1	0.0015	0.0646	0.8134
-0.0175	CLIP2	SCARF1	0.0021	0.0804	0.7653
0.0171	CLIP2	GADD45A	0.0027	0.0936	0.7575
0.0171	CLIP2	LOXL4	0.0027	0.0938	0.7575
0.0169	CLIP2	MAEA	0.0030	0.1004	0.7386
0.0166	CLIP2	COPS2	0.0036	0.1124	0.6934
-0.0164	CLIP2	SMAD6	0.0040	0.1207	0.6444
-0.0157	CLIP2	ADAM19	0.0058	0.1533	0.5920
0.0156	CLIP2	PPT2	0.0062	0.1592	0.5920
0.0155	CLIP2	RRM2	0.0064	0.1627	0.5920
0.0155	CLIP2	AURKAIP1	0.0064	0.1633	0.5920
0.0153	CLIP2	H1FX	0.0071	0.1730	0.5920
0.0153	CLIP2	ISOC1	0.0072	0.1741	0.5920
0.0153	CLIP2	PPIL3	0.0073	0.1758	0.5920
-0.0148	CLIP2	ANKRD52	0.0093	0.2030	0.5028
0.0146	CLIP2	RND3	0.0102	0.2142	0.5028
0.0146	CLIP2	ISCA2	0.0102	0.2144	0.5028
0.0145	CLIP2	RCAN1	0.0107	0.2210	0.4737
0.0145	CLIP2	HMGB3	0.0109	0.2226	0.4737
-0.0142	CLIP2	GLYATL1	0.0125	0.2414	0.4368
0.0140	CLIP2	RRP1	0.0141	0.2577	0.4198
-0.0139	CLIP2	CFH	0.0147	0.2647	0.3980
-0.0138	CLIP2	CD82	0.0157	0.2743	0.3980
0.0136	CLIP2	EGR1	0.0172	0.2875	0.3980
0.0133	CLIP2	SIRPA	0.0194	0.3063	0.3535
0.0130	CLIP2	RIMS2	0.0222	0.3289	0.3167
-0.0130	CLIP2	ANGPTL4	0.0225	0.3312	0.3167
-0.0129	CLIP2	SLPI	0.0240	0.3424	0.3167
0.0128	CLIP2	ANO2	0.0246	0.3464	0.3167
-0.0127	CLIP2	DUSP10	0.0254	0.3523	0.2801
0.0126	CLIP2	CENPH	0.0271	0.3639	0.2473
0.0126	CLIP2	EAPP	0.0275	0.3672	0.2471
-0.0124	CLIP2	SLC22A18AS	0.0294	0.3797	0.2261
-0.0124	CLIP2	PDE2A	0.0297	0.3814	0.2261
0.0123	CLIP2	CCBE1	0.0310	0.3903	0.2020
0.0122	CLIP2	LIMK1	0.0318	0.3960	0.0000
-0.0122	CLIP2	ZNF160	0.0322	0.3992	0.0000

SI Figure 2:

A) number of direct CLIP2 interactors in the GNA reconstructed from gene set 4, dependent on the edge probability cut-off in the *GeneNet* method. Red dashed line indicates the selected cut-off of 0.5

B) Edge-list of the 1st neighborhood of CLIP2 reconstructed from gene set 4.
pcor = partial correlation coefficient, node 1/2 = gene names of nodes,
pval = p-value of partial correlation test, qval = q-values (positive false discovery rate), prob = edge probability
black line: selected cut-off of 0.5

C)

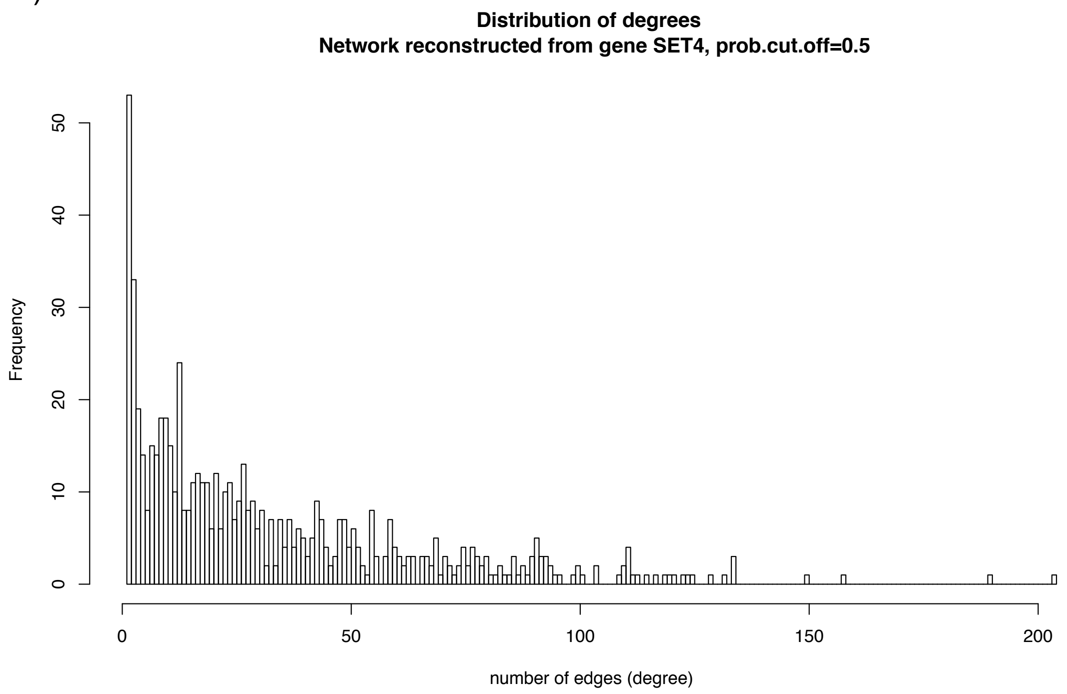

C) Degree distribution of the GNA generated from gene set 4 and a probability cut-off of 0.5 in the GeneNet reconstruction method. The degree distribution (approximately) follows a power law and thereby represents on attribute of a scale-free network.
