# Supplementary material for: Transcriptome network of the papillary thyroid carcinoma radiation marker CLIP2
Source: Radiat Oncol. 2020 Jul 29;15:182. doi: 10.1186/s13014-020-01620-5 (PMC7392692; doi:10.1186/s13014-020-01620-5)
Supplement: Supplementary file 3 — Additional file 3: SI Figure 3. Pathway enrichment analysis using the 50 Hallmark gene sets form MSigDB Fischer Exact Test was applied to test for enrichment of Hallmark gene sets within the 2nd-neighborhood of CLIP2 of the GNA reconstructed from gene set 4. 19 out of 50 gene set exhibited a significant enrichment with an adjusted p-value < 0.05 (red dashed line) [file 13014_2020_1620_MOESM3_ESM.pdf]

SI Figure 3

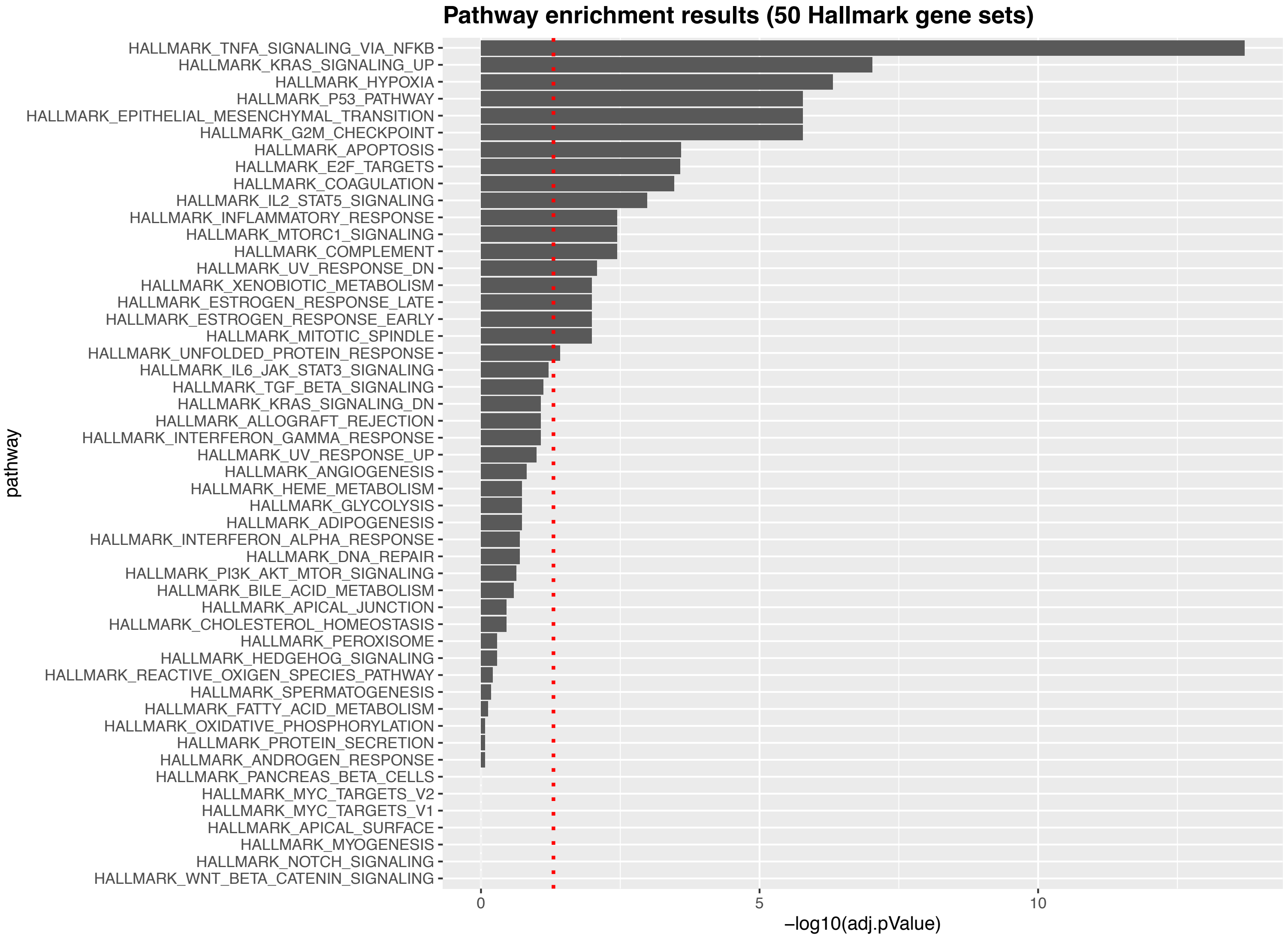

SI Figure 3: Pathway enrichment analysis using the 50 Hallmark gene sets form MSigDB Fischer Exact Test was applied to test for enrichment of Hallmark gene sets within the the 2nd-neighborhood of CLIP2 of the GNA reconstructed from gene set 4.
19 out of 50 gene set exhibited a significant enrichment with an adjusted p-value < 0.05 (red dashed line)
