# Supplementary material for: Transcriptome network of the papillary thyroid carcinoma radiation marker CLIP2
Source: Radiat Oncol. 2020 Jul 29;15:182. doi: 10.1186/s13014-020-01620-5 (PMC7392692; doi:10.1186/s13014-020-01620-5)
Supplement: Supplementary file 4 — Additional file 4: SI Figure 4. Validation of CLIP2 and PPIL3 protein co-expression by Immunohistochemical staining in papillary thyroid tumor tissue (10 cases) left column: CLIP2 staining right column: PPIL3 staining [file 13014_2020_1620_MOESM4_ESM.pdf]

SI Figure 4

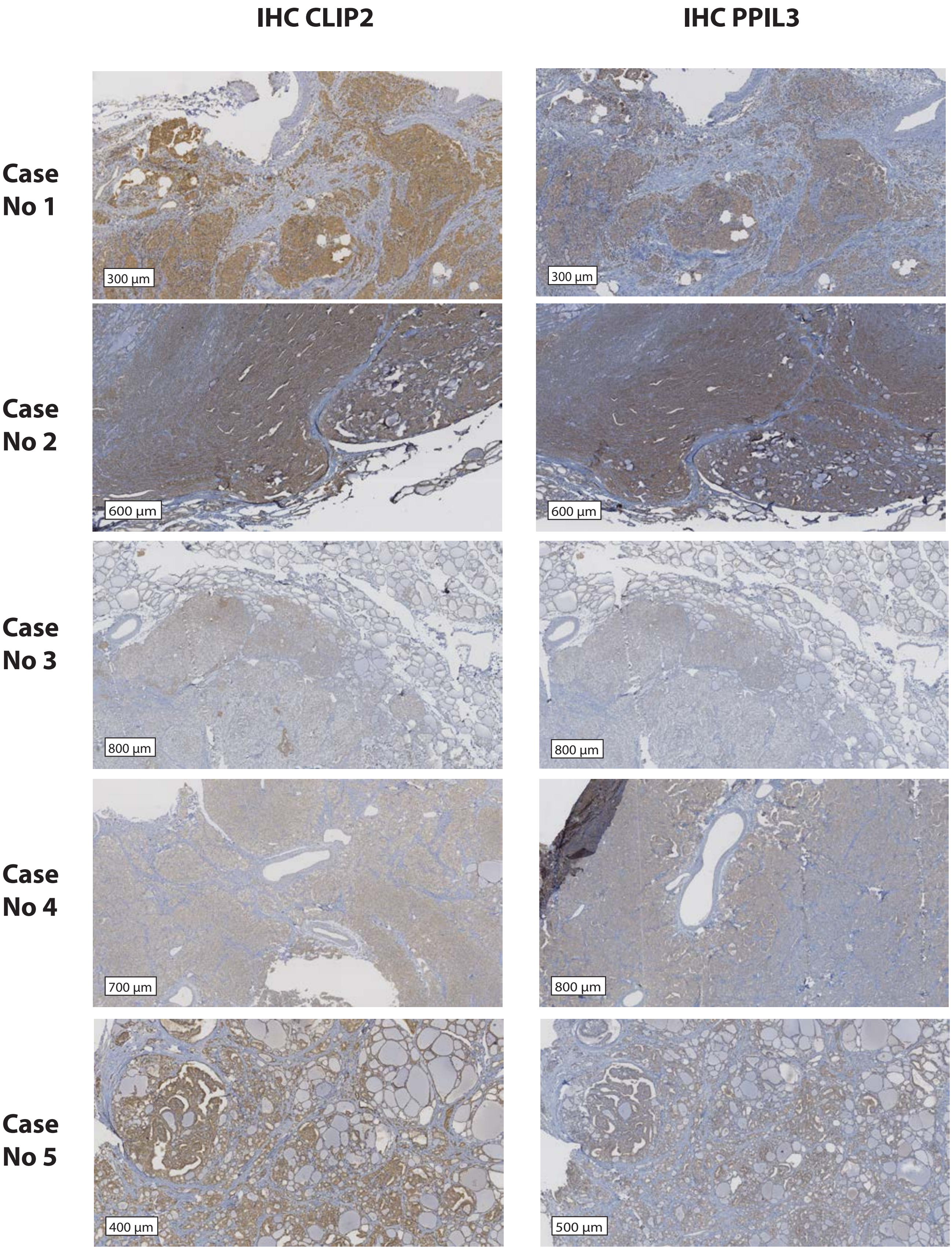
IHC CLIP2
IHC PPIL3
Case No 1
Case No 2
Case No 3
Case No 4
Case No 5
300 µm
300 µm
600 µm
600 µm
800 µm
800 µm
700 µm
800 µm
400 µm
500 µm

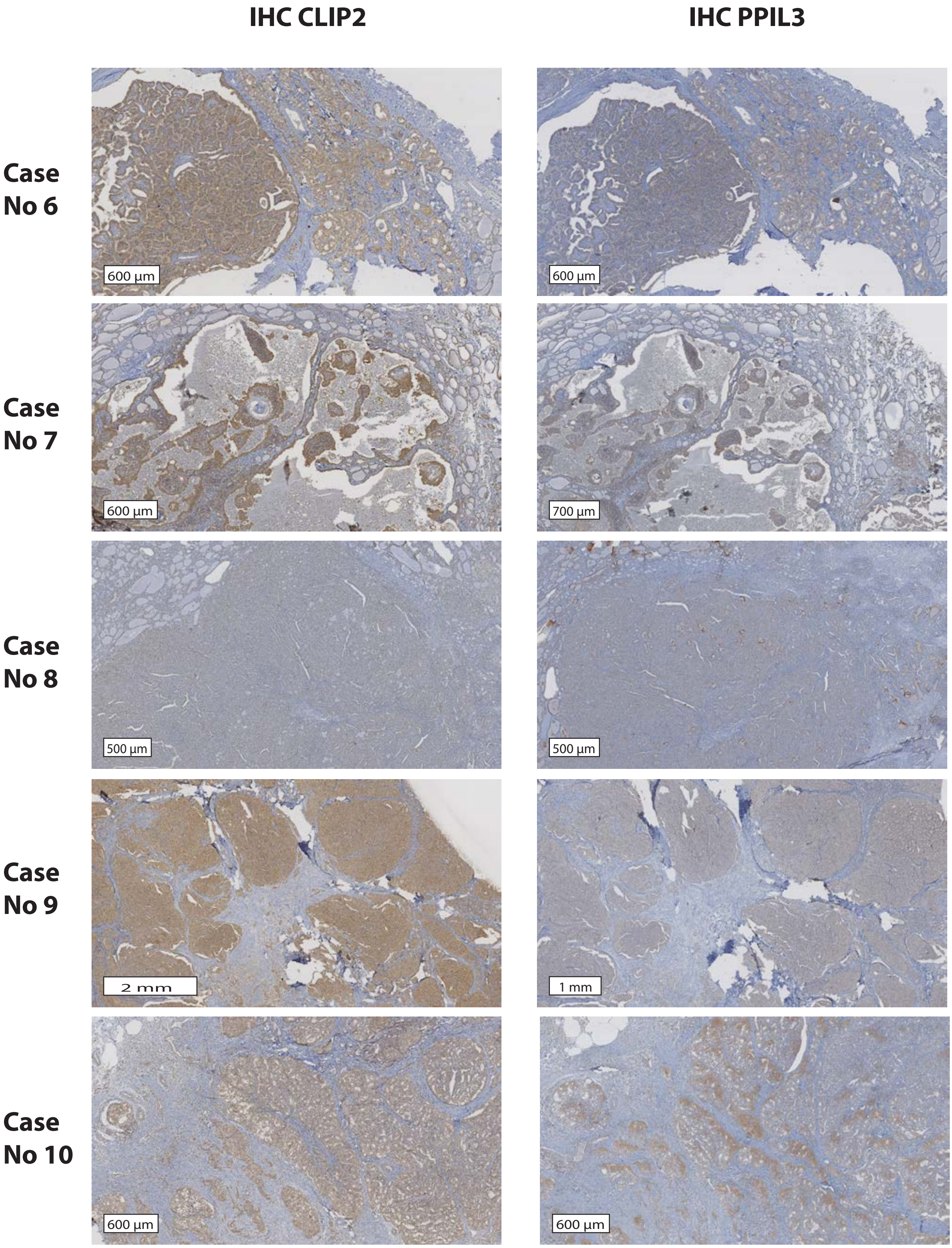

SI Figure 4: Validation of CLIP2 and PPIL3 protein coexpression by Immunohistochemical staining in papillary thyroid tumor tissue (10 cases)
left column: CLIP2 staining
right column: PPIL3 staining

Consecutive tissue sections, exhibiting similar histological structures were used.
