# Supplementary material for: Transcriptome network of the papillary thyroid carcinoma radiation marker CLIP2
Source: Radiat Oncol. 2020 Jul 29;15:182. doi: 10.1186/s13014-020-01620-5 (PMC7392692; doi:10.1186/s13014-020-01620-5)
Supplement: Supplementary file 5 — Additional file 5: SI Figure 5. First neighborhood networks of CLIP2 A) CLIP2 first neighborhood network extracted from BIOGRID database. Interactions of genes/proteins with a blue background color were derived from human biological material, while interactions with genes with a yellow background were derived from mouse biological material. B) CLIP2 first neighborhood network extracted from STRING database. Only interactions based of experimental data or co-expression are visualized. C) CLIP2 first neighborhood network reconstructed within this study D) CLIP2 first neighborhood network reconstructed from human patient gene expression data (papillary thyroid carcinoma) of the post-Chernobyl UkrAm cohort. [file 13014_2020_1620_MOESM5_ESM.pdf]

SI Figure 5: First neighborhood networks of CLIP2

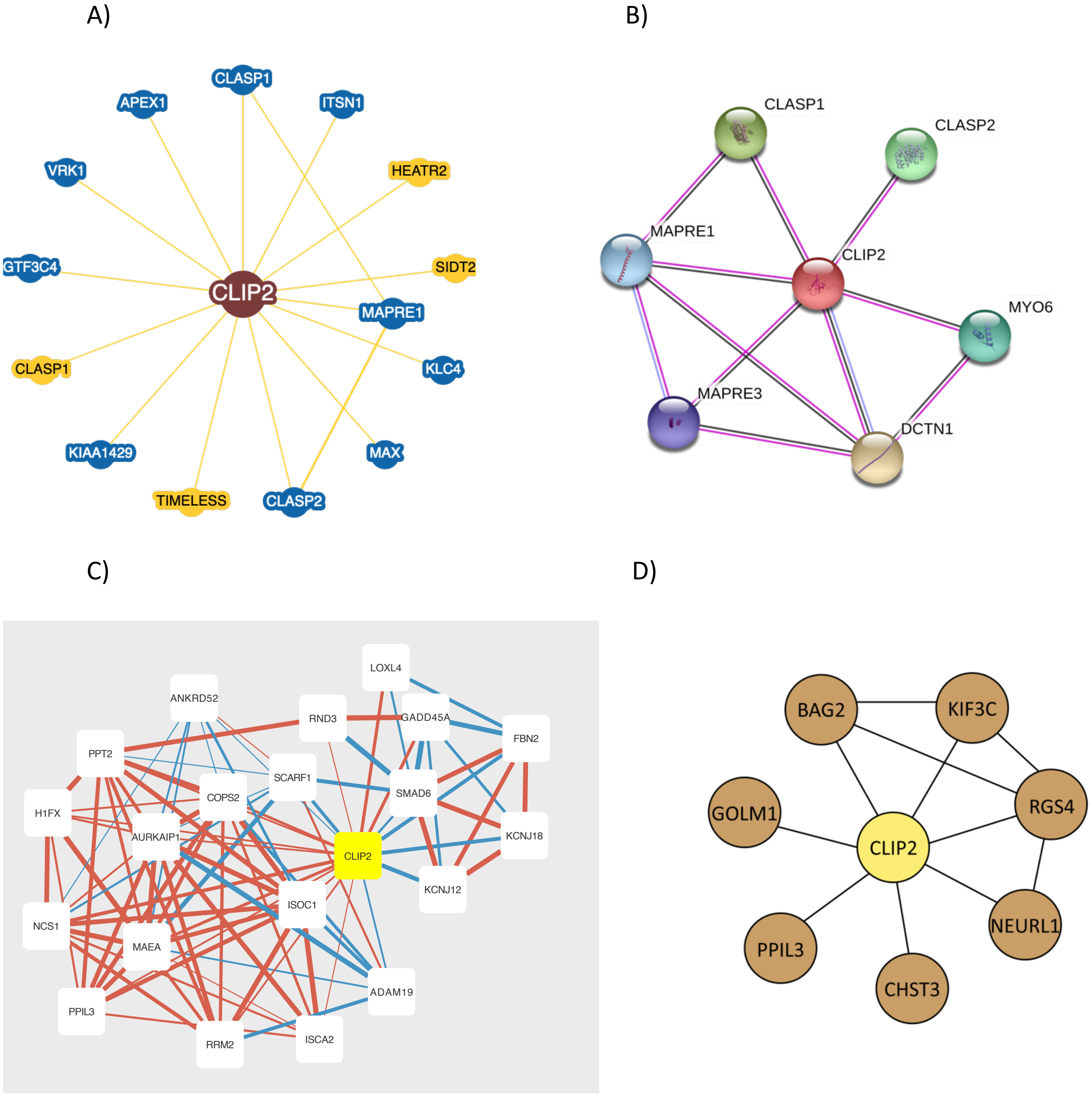

A) CLIP2 first neighborhood network extracted from BIOGRID database. Interactions of genes/proteins with a blue background color were derived from human biological material, while interactions with genes with a yellow background were derived from mouse biological material.
B) CLIP2 first neighborhood network extracted from STRING database. Only interactions based of experimental data or co-expression are visualized.
C) CLIP2 first neighborhood network reconstructed within this study
D) CLIP2 first neighborhood network reconstructed from human patient gene expression data (papillary thyroid carcinoma) of the post-Chernobyl UkrAm cohort.

Common CLIP2 interactions in A) and B): CLASP1, CLASP2, MAPRE1
Common CLIP2 interactions in C) and D): PPIL3
No common direct CLIP2 interactions in all other networks observed.
